# What influenced provision of non-communicable disease healthcare in the Syrian conflict, from policy to implementation? A qualitative study

**DOI:** 10.1186/s13031-018-0178-5

**Published:** 2018-11-12

**Authors:** Sylvia Garry, Francesco Checchi, Beniamino Cislaghi

**Affiliations:** London School of Hygiene and Tropical Medicine, London, UK

**Keywords:** Non-communicable disease, Syria, War, Conflict, Prioritisation, Public health, Implementation

## Abstract

**Background:**

There has been increasing focus on tackling the growing burden of non-communicable diseases (NCD) in crisis settings. The complex and protracted crisis in Syria is unfolding against a background of increasing NCD burden. This study investigated factors influencing implementation of NCD healthcare in Syria.

**Methods:**

This is a qualitative study, whereby semi-structured interviews were conducted with fourteen humanitarian health staff working on NCD healthcare in Syria.

**Results:**

Challenges to NCD care implementation were reflected at several stages, from planning services through to healthcare delivery. There was a lack of information on unmet population need; little consensus among humanitarian actors regarding an appropriate health service package; and no clear approach for prioritising public health interventions. The main challenges to service delivery identified by participants were conflict-related insecurity and disruption to infrastructure, hampering continuity of chronic illness care. Collaboration was a key factor which influenced implementation at all stages.

**Conclusions:**

The historical context, the conflict situation, and the characteristics of health actors and their relationships, all impacted provision of NCD care. These factors influenced each other, so that the social views and values (of individuals and organisations), as well as politics and relationships, interacted with the physical environment and security situation. Infrastructure damage has implications for wider healthcare across Syria, and NCD care requires an innovative approach to improve continuity of care. There is a need for a transparent approach to resource allocation, which may be generalisable to the wider humanitarian health sector.

**Electronic supplementary material:**

The online version of this article (10.1186/s13031-018-0178-5) contains supplementary material, which is available to authorized users.

## Background

Non-communicable diseases (NCDs) were responsible for 39.8 million deaths worldwide in 2015 [1]. Most of NCD deaths are caused by cardio-vascular disease (CVD), diabetes mellitus (DM), chronic respiratory disease and cancer [2]. Rising NCD rates are partly attributable to an ageing population, but also to changes in environmental and lifestyle behaviours including obesity, smoking, physical inactivity and alcohol consumption [2]. NCD rates and associated risk-factors have been steadily rising across the Eastern Mediterranean region in recent decades [3–5]. NCD prevalence in Syria has increased since 2000, with an estimated 40% of adults now at risk of CVD and DM [6]. NCD-related mortality in Syria is also rising, a trend maintained when standardised for age [7, 8].

Pre-conflict Syria had some of the best regional health indicators [4, 9] and produced more than 90% of its medications in-country [9]. However since the Syrian War began over 7 years ago, this picture has drastically changed, with 13.5 million people now in need of humanitarian assistance [10]. By 2015, 4 in 5 Syrians lived in poverty; and by 2016, almost 1 million people were in besieged areas, suffering restrictions to healthcare, food and water [10]. Only half of aid convoys reach the population due to blockades [10]. Healthcare facilities have been attacked [11] and by June 2017, only 49% were fully functioning [12]. Humanitarian health responses for Syria were coordinated through UN-activated clusters [13] with three geographic hubs (Damascus, Turkey and Jordan) at the time of this study. Healthcare in opposition-controlled areas was mostly provided by local healthcare workers, supported by remotely by non-governmental organisations (NGOs) [14].

Conflict increases the need for healthcare, while reducing health system capacity [15]. NCDs have been increasingly recognised as a problem in conflict areas but this is still a new area of work [16–19]. The UN Interagency Task Force (UNIATF) [20, 21] produced a strategy for NCD care in emergencies [22]. However, providing NCD care requires not only understanding the burden and technical guidelines [23, 24] but also a reliable supply of affordable medications, and access to trained healthcare workers and equipment. Reduced access to healthcare and disrupted pharmaceutical supplies cause treatment interruptions [17, 25, 26]. Evidence on effectiveness and cost-effectiveness of NCD interventions in crises is limited [16, 19] and resource allocation is a challenge in these contexts [22] due to the immediacy of competing needs, limited resources, and lack of focus on NCDs in emergencies by both donor agencies and healthcare providers [17, 18].

Currently 6.3 million people are displaced within Syria [10] but information on the health of Syrians within Syria is limited [27]. Internally-displaced persons (IDPs) are particularly vulnerable even when compared to refugees, with increased mortality [28] and fewer protection mechanisms [29]. We wished to explore factors impacting provision of healthcare in conflict settings by using NCDs during the Syrian War as a case-study. In this context, care to opposition-controlled areas is mostly supported by the humanitarian response system, as outlined above. Therefore we focused on factors influencing this pathway, from policy to implementation. Such insight could help healthcare providers at national and international levels plan responses [24]. We carried out a literature review to further contextualise the references. For brevity’s sake, the methods and results of this review are presented in Additional file 1. A qualitative approach was used to gather data from a range of stakeholders involved in NCD healthcare strategy and delivery.

## Methods

A semi-structured interview approach was used to allow flexibility and facilitate open discussion. We elected against focus groups as this method could have discouraged participants from freely expressing views, particularly in a context where security concerns limit information sharing [30]. We developed an interview guide in consultation with academics and NGO workers, to include open questions around challenges in NCD care delivery, resource allocation, funding and collaborations. We piloted it for clarity and appropriateness with a small group of participants, and refined it based on their feedback.

Participants (*n* = 14) had worked in Syria with the World Health Organisation (WHO) (3 participants), for international NGOs (iNGOs) (6 participants) or were Syrian health actors in opposition-controlled or contested areas (5 participants) (Additional file 2: Table S3). Participants gave written consent to participate in the research and all data were made anonymous. The interviews were conducted by Skype during June–August 2017 in English, and one in Arabic via an interpreter. Interviews were recorded, transcribed, and uploaded onto NVivo © software (v11). Thematic analysis methods were used [31–33], whereby data were compiled, disassembled and re-assembled [31]. The thematic analysis method was used as it allows themes and patterns to emerge from the data and can inform policy implementation [32]. This analysis started from the first interview, so that data collection and analysis were iterative. Data were coded by allocating phrases and sentences to nodes and sub-nodes. Sub-nodes were grouped together to re-assemble the data into a hierarchical array with the primary data being consulted to ensure themes were representative.

**Fig. 1:**
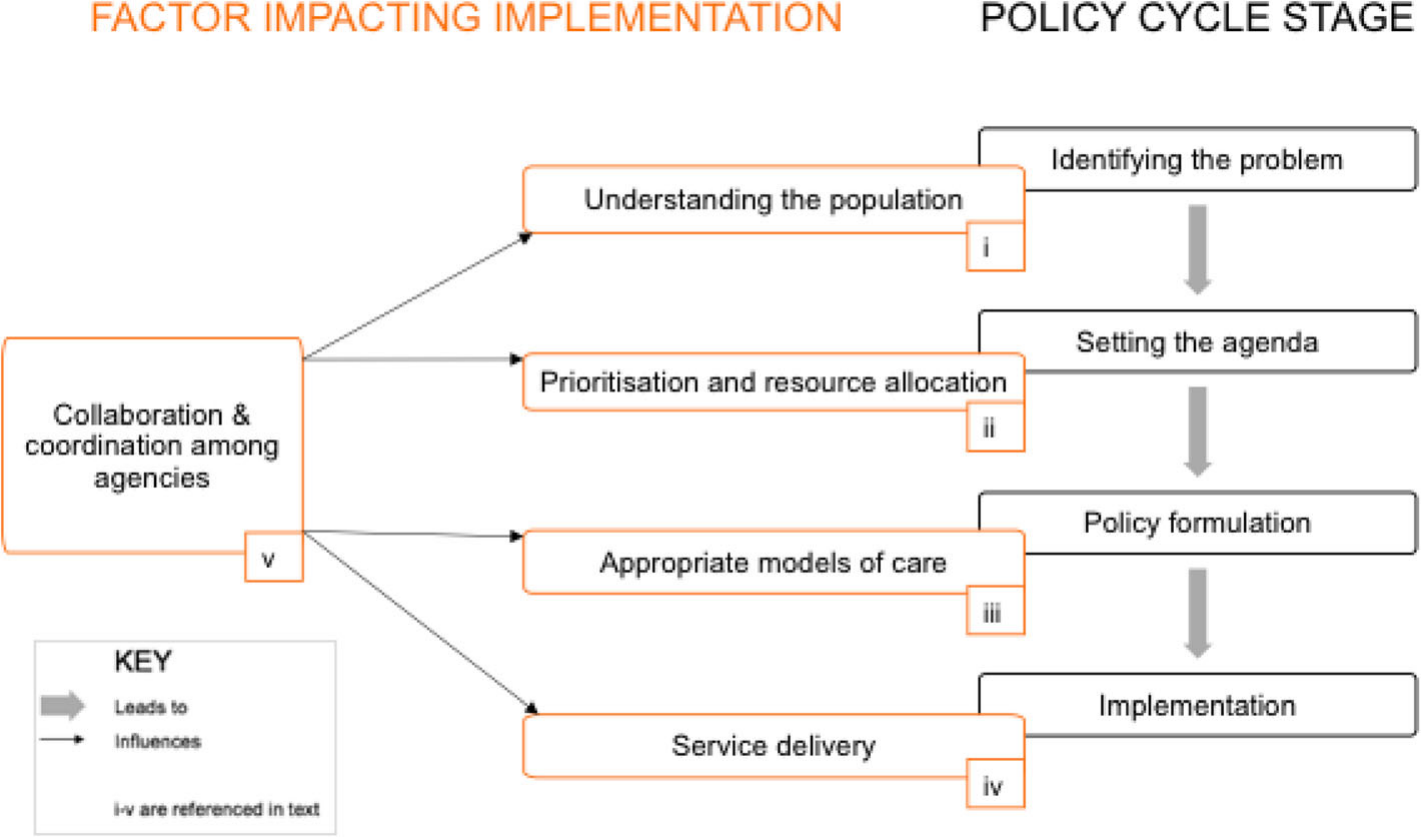
Study results: challenges impacting NCD care in Syria

Previous researchers have used models to categorise factors influencing policy creation, adoption and adaptation, including Walt and Gilson’s framework [34–36] or Kingdon’s agenda-setting streams [37]. The framework for this study (Fig. 1) was developed using a bottom-up approach based on key themes emerging from the data, compared to these existing frameworks in the literature. Emergent themes from the analysis were mapped onto five key sequential areas of challenge in the cycle, from policy-setting to implementation (Fig. 1): understanding population need; prioritisation; determining appropriate healthcare models; service delivery; and collaboration and governance.

## Results

### Challenge 1: Understanding the population need for NCD care

The first challenge to emerge was identifying the problem itself (Fig. 1), as there was a lack of consensus on the need for NCD care compared to other health services. There are several components to health needs: the population’s perceived needs, both expressed and not expressed; the professionals’ perspectives on what a population needs; and a relative health need, based on what is known about other populations [38]. Participants reported a lack of population level data, and interviews revealed disagreement about how to allocate resources among different health services, especially between care for war trauma and other urgent cases, versus treatment for chronic disease.

#### Perception of population need

One international participant recognised the population did not adequately identify their own needs: *“there’s a lack of knowledge […]; how do [patients] know that [they’re] not well as some NCDs are silent?”* Such unawareness would reduce demand for NCD services.

There was no cohesive health information system analysing NCD morbidity and mortality. Needs assessments were based on incomplete service data and HeRAMS (Health Resources and Services Availability Monitoring). Several participants from all backgrounds described the lack of patient voices. One international participant reported: “*There’s this assumption made about the population’s health solely from the health facilities, so it’s already biased.”* One international participant added that *“we have also been thinking about people who are housebound, people with mobility issues, those invisible people.”* A WHO participant stated that *“culturally…the patient voice is not very much heard.”* This made it difficult to understand unmet population need.

Many participants felt projects were donor-driven rather than needs-driven, with a WHO participant suggesting that *“this is more a provider-driven system than a response”*. An international participant described a gap between what donors would fund, and population needs: *“A Syrian NGO recognised NCDs was a problem, but said the donors won’t give funding.”* Overall service planning was described as a predominantly top-down approach, rather than based on population need.

#### Trauma and infectious disease perceived as the pressing needs

Participants, especially those working locally, felt that injuries and control of infectious diseases were generally treated as the priority public health needs. Infectious disease was concerning due to the potential for spread, with a local participant reporting *“fear of communicable diseases like cholera and polio.”* Most with experience as healthcare workers (HCW) inside Syria regarded injuries as the greatest need, saying: *“We were consumed with war injuries”.* This was due to their immediacy (*“when you have airstrikes, many people are injured and need to go to hospital, you focus on this issue”)* and visibility (*“the view of blood attracts attention”).* Many participants agreed promoting healthy lifestyles was not top of the donor and healthcare providers’ agendas, with a focus on more immediate needs. One local participant reported, referring to NCD care: *“you know [because of] trauma and war, you cannot pay attention to these issues.”*

Participants at all levels described a separation between emergency care models, and long-term approaches to health and development. One international participant believed that *“Syria is no longer an acute emergency, it’s not in the development period. It’s in the middle bit, where you have a failed health system.”* All international participants reported the challenging dichotomy of providing chronic care in an emergency setting, with one WHO participant saying: *“acute emergencies and chronic disease are like oxymorons, they are opposing terms.”* This was also reflected in donor attitudes, which were described as focusing on emergency aid, with one international participant reporting: *“Some donors think in health emergencies we do not support advanced healthcare or structuring health systems.”*

#### Lack of consensus on need for NCD care

Participants disagreed about the long-term benefits of NCD care. One international participant suggested other health services were more cost-effective: *“You can reach thousands of children quickly and at a lower cost than managing a few thousand NCD cases.”* A WHO participant agreed: *“Talking about population-based measures in situations of crisis, is not the right time for that. […] Business as usual doesn’t work.”* However, this was not universal. An international participant reported: *“When they sent back data we realised, wow, people are actually seeing cases and we’re not paying attention to it.”* Another international participant reported frustration with the default assumption of health priorities: *“The vast majority of funds, on the level of millions of euros, goes for trauma. This has been plaguing me.”*

A local participant reported the challenge of creating continuity of care contrasted with the brevity of funding: “*Most funding is one year or six months, you would not be able to develop anything.”* Therefore it was challenging to plan for long-term risk reduction strategies for patients when organisations in these contexts classically focused on emergency care and immediate health gains.

Overall there was no consensus on the population need for NCD healthcare, and multiple other competing priorities for this population.

### Challenge 2: Prioritising needs within NCD care

The second challenge to emerge was prioritisation within NCD care (Fig. 1). Prioritisation is a systematic approach to allocating limited resources. This process of ranking needs is complex and requires understanding population needs. Funding allocation in crises is complex, and is often directly linked to specific contexts or objectives. At the global level, NCD-specific funding is difficult to measure as it is mostly subsumed within general healthcare funding [39].

#### There was no prioritisation framework for NCD healthcare

NCD care is often incorporated into programmes at the primary care level. However, international participants reported a lack of local knowledge and a lack of coordinated overall strategy: *“they don’t have a clear strategy on how approach these NCDs on a national level”*. There was universal frustration with the lack of a clear, transparent decision-making framework for explicit and fair resource allocation. A WHO participant summarised:

*“What is missing is how we decide on resource allocation, who decides. There are no clear standards about how to prioritise NCDs. If you don’t say how you do it, there is no appeal process. It becomes unfair and difficult to accept.”*

Several participants advocated for a cross-cutting prioritisation framework across all health areas, which one WHO participant expressed as requiring *“a more comprehensive approach [rather] than a piecemeal disease-specific one.”*

#### Should there be limits to care?

Prioritisation requires challenging and important conversations regarding benefits and harms of actions and inactions, including discussions on limits to care. Whereas the majority of local participants did not agree that limits to care should be accepted, there was a wide range of uncertainty expressed by the international community. One WHO participant reported conflict in the international community: “*I have colleagues saying there is no place for cancer in conflict settings. I say …. these are amongst the sufferings. We might decide what action might be taken, even palliative care.”* Another international participant reflected: *“In conflict situations, people with chronic kidney disease are going to die. Don’t spend money on dialysis, you have a limited pot of money.”*

Overall, there was no agreement on priorities within NCD healthcare by participants, and how to approach the prioritisation process in a crisis setting. Allocation of resources between and within health services was not made transparently.

### Challenge 3: Determining appropriate models of NCD healthcare delivery

The next challenge uncovered was how to deliver healthcare (Fig. 1). The interviews revealed some consensus: that best-practice management of individuals (such as which medication should be used to treat hypertension or diabetes) could generally be agreed; that NCD care should be integrated horizontally into healthcare systems; and that effective NCD management requires continuity of care. However, the data also pointed to a mismatch between opposing approaches to healthcare. NCD care can broadly be divided into three levels: primary prevention focuses on averting occurrence of disease; secondary prevention aims to identify and manage illness amenable to treatment; and tertiary prevention aims to reduce complications [40]. International stakeholders favoured a focus on primary and secondary prevention, whereas local participants favoured specialist care to treat illness and manage complications.

#### The international model: Prevention

The international model focused on delivery at primary care or community level. All participants agreed that the conflict environment created specific local challenges for implementing primary prevention strategies. As one international participant described: *“People smoke more because they’re stressed and bored, …. healthy food is expensive and not available… going out jogging every morning is not top on your list of priorities.”* One international participant highlighted the lack of awareness of the importance of early management of illness: *“People don’t recognise why they’re treating hypertension, [they] don’t think about what they’re doing, that they’re trying to prevent heart attacks and strokes and kidney failure.”* As another international participant working with local HCWs explained: *“Working in the north of Syria, after 6 years, the conversation is the same, there was a total movement away from primary care.”* In general, international participants reported frustration that the benefits of prevention were not appreciated locally.

#### The local model: Specialist care

The local model was based on delivery at the specialist level. Most participants felt referral routes for specialist care across borders were problematic, and an international participant described: *“The process of referral to Turkey is really hard. The referral mechanism has a long process.”* However, whereas international participants aimed to strengthen this referral route, local participants wanted to strengthen the in-country provision of specialist care, saying: *“many NCDs need tertiary units, and tertiary services”*. They expressed frustration at the lack of access to diagnostic tests and treatment, reporting that this was the main priority. Some international participants felt this focus on in-country specialist services was excessive, as one international participant described: *“partners on the ground want their toys, they want big diagnostic equipment, they want it to be how it was before the war.”* This was a marked difference in attitudes towards specialist care.

#### Acceptability of healthcare delivery

Participants agreed that the model of pre-conflict healthcare heavily influenced current population expectations, and the acceptability of healthcare models offered. Local participants described how in pre-conflict Syria, patients directly sought specialist care and did not consult with a generalist:

*“There was no system where you start by a general practitioner and then be referred to secondary and tertiary. Whenever people have health issues, they go to a cardiac specialist doctor. That was happening before, it’s still happening now. That’s how Syrian people are.”*

This was mirrored by international participants. A basic package of NCD care includes generic medications, as these are cheaper than branded equivalents. An international participant described the difficulties in delivering standardised approaches: *“We tried to match their medications, but they’d say “no, we were taking the blue pill before. This one, it’s not blue, I want the blue pill.””* These expectations reduced the acceptability of delivering NCD care in primary care.

The model of healthcare delivery, via primary or specialist care, was an area on which participants had clear opposing views.

### Challenge 4: Delivery of NCD services

The Syrian conflict has caused unpredictability, insecurity, political discord, and infrastructure collapse, resulting in challenges to resource distribution and access. As such, this creates a challenge to implementation (Fig. 1) by impacting logistics of running services, as well as access to services.

#### Volatile security necessitates flexibility

Syria is a complex emergency, with varying health needs across locations. A WHO participant explained this complexity: *“It is not simply a conflict, or post-conflict or transition state. The needs change from month to month on a short time scale.”* As a WHO participant summarised: *“You know, there is not one Syria, there are many Syrias within Syria.”* International participants described variable access to healthcare and disruption to the infrastructure: *“There are good days and bad days, life goes on. Staff would tell me “my cousin had a wedding last week”, in other areas they’re living in a bunker.”* Some participants described difficulties of maintaining continuity of care to a mobile population, and how healthcare needs changed; flexibility and responsiveness in service delivery and resource allocation were therefore required.

All participants agreed NCDs should be horizontally integrated into healthcare. Several international participants emphasised the need to build resilience, with a WHO participant reporting: *“Redundancy is important. [If] you have a central facility managing a condition or a group of patients, and it’s the only one, it becomes difficult to continue care.”* All agreed that NCD healthcare provision requires system resilience.

#### Conflict impacts logistics of service delivery and access

Unsurprisingly, all participants agreed security was a major determinant of healthcare delivery, with a WHO participant reporting: *“In besieged areas, the main determinant becomes conflict itself, and an inability to access because the facility has been destroyed, the providers are no longer there”.* Local participants reported this also impacted and reduced provision of medications from the black market. All reported challenges around resources such as equipment and medications, with one international participant summarising the situation: *“So much of the issue is the infrastructure, the laboratories, the reagents, the supply of medicines, the supply of sphygmomanometers and stethoscopes.”* Insulin was of particular concern, with difficulties around procurement and supply, as reported by one local participant:

*“We depend on imported insulin; […] we always have this concern of not having enough insulin, not having it on time, not having it in the proper way, like transporting with refrigeration.”*

#### Providing healthcare in conflict is dangerous and expensive

Security was a huge challenge due to damage to healthcare structures and deaths of HCWs, as one WHO participant described: *“The main determinant is the war itself…. there is no effective assistance without protection.”* Many participants described HCWs and healthcare structures being directly targeted. One local participant said: *“A lot of staff are tortured, killed or left the country.”* As one international participant explained:

*“We were humbled and tearful at these extraordinary people doing a job under such difficult circumstances. They say, clinic gets bombed and buildings destroyed, we’ll send in a mobile clinic. The doctors have got killed, we’ll send in medical students.”*

Additional resources were also required to improve security, as a local participant explained: *“establishing underground hospitals, fortifying hospitals, due to direct targeting of all facilities; this consumes considerable amount of money.”* This would increase the resources required to provide healthcare in this context.

Conflict impacts service provision, but also endangers lives of HCWs in this environment, which creates a moral quandary as providing health may increase risk for HCWs.

### Challenge 5: Collaboration and governance

Finally, participants highlighted how the interactions and relationships among stakeholders impacted all other challenges outlined (Fig. 1). Healthcare and war clearly have competing objectives, and there was widespread agreement regarding the complex challenges this brings, including clear leadership, trust and power dynamics.

#### Power dynamics with the regime

All participants described political complexity of working together, or at least cooperating, to provide healthcare. This was about perception and fear of repercussions. A local participant explained: *“There is very little interaction with the MoH [Damascus Ministry of Health] [...] This is a very sensitive relationship, it is very sensitive to talk about.”* This impacted logistics, as an international participant described: *“At the UN level, the challenges are related to [being] willing to be identified to be working in opposition areas.”*

There were several reports of enforced blockades resulting in healthcare provisions not being allowed into besieged areas. One WHO respondent described: *“The only one who can provide the necessary medications to the besieged area is Damascus.”* Some participants felt healthcare provision was explicitly used to give the appearance of power and control, with one international participant saying: *“The Damascus MoH try to prove they have access to opposition-controlled areas. And they don’t, but this is perceived as a political intent to show control.”*

#### Leadership

Local Directorates of Health (DoH) emerged to provide health leadership in the non-governmental-controlled areas, initially in an informal capacity. Participants disagreed regarding the effectiveness of this leadership. Local participants reported growing trust and reliance on the DoH: *“There were no governance bodies, no health authorities, no government. The health directorate was gradually playing an important role in the governance of the health sector.”* However, some international participants had concerns, with reports of weak leadership, and of an agenda to gain power and recognition. One international participant reported:

*“You have ineffective and disrespected DoH. They want control over the whole environment. They get heavily involved and they put their names out there, so they can get power and recognition and funding. They’re not actually delivering the services where they are most needed. Eventually they want to be part of a new Syrian anti-regime MoH.”*

#### Governance and corruption

The consequence of disrupted governance was corruption. Many participants reported incidents of corruption, with a local participant saying: *“The corruption was real unfortunately.”* The scale of corruption was felt to be significant by international participants: *“There’s more corruption in this environment than I’ve ever seen in the civil society types of movements elsewhere.”* This has resulted in restrictions to funding, programmes and negatively impacted on healthcare programmes.

Participants strongly felt that healthcare was used to try to alter power dynamics.

## Discussion

### Findings

**Fig. 2:**
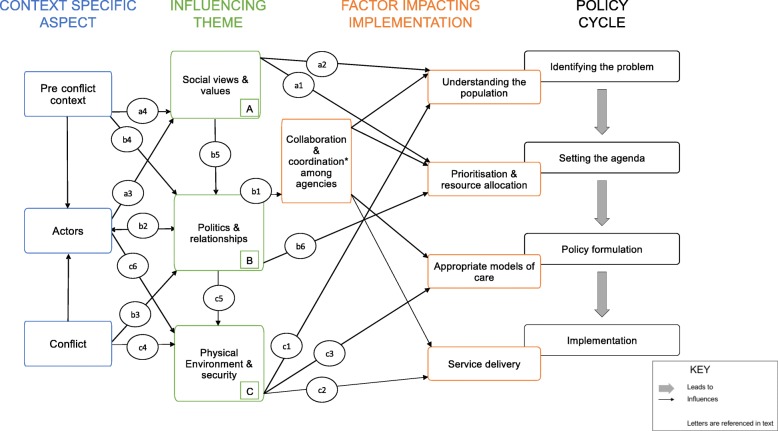
A model of barriers to NCD care in Syria: factors impacting NCD care implementation, and the upstream aspects and themes that lead to these factors

Our study described factors directly influencing NCD healthcare provision, by impacting policy creation and healthcare implementation at several levels. Figure 2 shows key upstream aspects that emerged as reasons for these factors: the pre-conflict setting, actors involved, and the conflict itself. The actors include international and local agencies and individuals, and the population. These upstream context-specific factors are not independent from each other. The causal relationship between these and factors impacting implementation are mediated by three key themes (A, B and C in Fig. 2) as explained below, with the references in brackets (e.g. a1) relating to those in Fig. 2.

#### Social views & values (A)

Social views and values refer to how individuals, populations and organisations see the world, and determine what is most important. NCD healthcare provision within Syria is dependent on defining the scope of the problem and setting the agenda (a1). Defining population need is value-driven (a2), since it depends on what health means [41]. NCDs compete with other NCDs as well as other health and non-health priorities. Trauma care was prioritised, as seen elsewhere [42]. We have shown the challenges in gathering a unified vision, with shared goals and objectives. This is a reflection of different world perspectives, different approaches to health priorities, and different understanding of what societies value.

Top-down and bottom-up factors influenced prioritisation. Bottom-up influencers were the population voice, direct fieldwork experiences, and ground needs assessments. HCWs spoke emotively of the population’s daily struggles, echoing descriptions elsewhere of competing priorities due to loss of shelter and income [10]. There were references to the “invisible” population of Syria, who do not seek healthcare themselves, who may be isolated or stay at home. This includes less mobile people such as the elderly and those with physical disabilities. The lack of input from beneficiaries, especially from this “invisible” population, was clear. Top-down influencers were donors, with inflexible frameworks for funding. Participants reported these were the most influential in determining priorities. International participants were constantly aware of mandates and agendas, funding, and models of healthcare used elsewhere.

People’s values reflect training and experiences (a3). The pre-conflict healthcare system influenced both HCWs and the beneficiaries (a4), which was reflected in expectations of receiving care and priorities in care. This exemplifies the importance of understanding the pre-conflict context.

#### Politics & relationships (B)

Political interactions and relationships amongst individuals and agencies impact all areas of NCD healthcare provision, through collaboration and coordination (b1). These relationships are complex, including opposition and government-controlled areas; international communities and other countries; and between and within international agencies. The delicate nature of these interactions was clear throughout (b2).

Syria is at war, in a struggle for power (b3). This dynamic process had periods of variable stability and collaboration, influenced by pre-conflict relationships (b4). Healthcare became a political tool at all levels, international, local, and even as part of the conflict.

The agendas of organisations (b5) and lack of transparency in decision-making processes hampered collaboration and created a power imbalance between funding agencies and providers. The link between funding and power was evident throughout as the funding agencies’ power pervaded discussions due to their influence on agenda and vision (b6). The scramble for funding often overshadowed needs-based assessment.

Challenges to governance are described elsewhere, due to the presence of multiple actors, including the Damascus regime, opposition forces, local councils, and the Islamic State [29]. The impact of ambiguous governance is evident through corruption incidents, and challenges to quality control. The latter is magnified by the need to work remotely in besieged areas.

#### Physical environment & security (C)

Security influences multiple processes, including gathering data (c1), planning services, and implementation (c2). This is the largest determinant of service delivery due to chronic infrastructure collapse leading to shortages of HCWs, medicines and equipment. Conflict impacts on models of care (c3) since continuity of care and healthy lifestyles become more challenging in displaced populations. The physical environment and security are, however, dynamically dependent on the conflict’s evolution (c4). This area is complex: security is dependent on politics and conflict (c5), but is influenced by health actors (c6) through témoignage and advocacy. Additional resources are required for healthcare structure fortification, increasing the pressure on already limited resources. The lack of reliable access creates further challenges for continuity of care.

NCD-related mortality is rising, accounting for almost 69,000 deaths in Syria in 2015 [43], half of which were in people younger than 70 years [43]. Refugee studies have reported that 1 in 5 Syrian refugees have at least one NCD, with a quarter unable to seek care [44]. Unmet health needs of those in country are likely to be high due to vulnerable populations [45]. WHO estimates that 80% of CVD and DM can be prevented through risk-factor modification [43]. However NCDs do not gain the same attention as other more immediate needs [18]. The UNIATF brief does not outline an approach to prioritisation [22]. Stakeholders had different views and services were based on perceived need. The lack of consensus on benefits of long term care made it difficult to achieve consensus on the appropriate package of services. Our findings re-affirm this by highlighting disconnect between viewpoints of international and local stakeholders, and the disagreement regarding balancing immediate needs versus long-term health investment.

### Study limitations

Participants in the interviews mostly worked in opposition-controlled areas, which is a source of selection bias. Interviews are two-way processes, and the interviewer (SG) has experience of working as a front-line healthcare worker in conflict settings; this would inevitably have some impact on the responses. Care was taken during the interviews to use open questions and neutral responses to minimise this. In some cases, the participant may not have been willing to speak honestly about their experiences, or be seen to criticise organisations. Using an interpreter for the Arabic speakers was particularly challenging, as some intricacies of the questions were lost.

While our study examined challenges in policy-making and implementation, it did not capture beneficiary perceptions of actual access to and quality of healthcare. Moreover, we considered that our qualitative approach would be less useful for documenting actual service performance (availability, coverage, quality), for which objective indicators would be more informative.

## Conclusions

The historical context, current situation, actors and their relationships, all interacted to affect NCD care in Syria. Our findings can help explain challenges encountered, and plan on how to overcome these.

The NCD response in Syria does not fit neatly into either the UNIATF (UN Interagency Task Force on NCDs) “initial” or “continuing” response scenarios [22]. The “initial” approach focuses on emergency rather than continuity of care, and the “continuing” approach is challenging to implement in a context with unpredictable security. Implementation of NCD care requires flexibility and contextual insight.

Defining the problem requires a coordinated approach between agencies to share data [11], which would be facilitated by standardising information collection methods. The beneficiaries’ voice was largely absent: service planning should involve the population at all levels [46]. Working closely with local actors and social scientists would improve this understanding.

Further evidence is required regarding the long-term benefits of NCD care in conflict situations, including cost-effectiveness. Challenges in prioritisation in crises are not new [47] and a transparent approach is required to prioritise across health service areas (e.g. mental health, vaccination, nutrition) and within areas, e.g. oncology. This paper calls for a resource allocation framework for ethical and fair distribution [48] to improve accountability and equity. Such a framework should not be unique to NCD care or Syria, and instead be adopted globally.

## Additional files

Additional file 1: **Table S1.** Literature review on factors impacting NCD care in Syria: search criteria. **Figure S3.** Literature review: articles identified and number of articles excluded at each stage, adapted from PRISMA [44]. **Table S2.** Literature review: overview of factors impacting NCD healthcare implementation in Syria. (DOCX 47 kb)

Additional file 2: **Table S3.** Overview of participants interviewed. (DOCX 16 kb)

## Abbreviations

CVD: Cardio-vascular disease
DM: Diabetes mellitus
DoH: Directorates of Health
HCW: Healthcare workers
HeRAMS: Health Resources and Services Availability Monitoring
INGO: International non-governmental organisations
MoH: Ministry of Health
NCD: Non-communicable disease
UNIATF: United Nations Interagency Task Force on NCDs
WHO: World Health Organisation
