# Supplementary material for: What influenced provision of non-communicable disease healthcare in the Syrian conflict, from policy to implementation? A qualitative study
Source: Confl Health. 2018 Nov 12;12:45. doi: 10.1186/s13031-018-0178-5 (PMC6233508; doi:10.1186/s13031-018-0178-5)
Supplement: Supplementary file 1 — Table S1. Literature review on factors impacting NCD care in Syria: search criteria. Figure S3. Literature review: articles identified and number of articles excluded at each stage, adapted from PRISMA [44]. Table S2. Literature review: overview of factors impacting NCD healthcare implementation in Syria. (DOCX 47 kb) [file 13031_2018_178_MOESM1_ESM.docx]

Appendix 1

*Table 1: Search criteria used for literature search on factors impacting NCD care in Syria*

|  | **Search terms** |
| --- | --- |
| **1** | ((armed or zone) adj2 conflict$).mp. |
| **2** | (conflict adj3 (population$ or person$ or communit$ or people)).mp. |
| **3** | (displaced adj3 (population$ or person$ or communit$ or people)).mp. |
| **4** | (evacuate* or IDP*).mp. |
| **5** | War exposure/ |
| **6** | war.mp |
| **7** | 1 or 2 or 3 or 4 or 5 or 6 |
| **8** | syria*.mp. |
| **9** | Syria exp/ |
| **10** | 8 or 9 |
| **11** | (Non communicable disease* or Non-communicable disease* or Noncommunicable disease* or NCD* or chronic disease* or chronic condition* or long term condition* or heart disease or cardiovascular or cerebrovascular or stroke or hypertensi* or cholesterol* or cardiac or myocardial infarct* or coronary heart disease or CHD or blood pressure or blood sugar or blood glucose or diabetes or obesity or cancer* or neoplasm* or asthma* or respiratory or COPD or chronic obstructive pulmonary disease* or pulmonary or chronic kidney disease* or CKD or renal failure or public health or smok* or diet).mp. |
| **12** | exp non communicable disease/dm, ep, pc, rh, th |
| **13** | chronic disease/ or exp multiple chronic conditions/ |
| **14** | exp public health/ or exp social medicine/ |
| **15** | 11 or 12 or 13 or 14 |
| **16** | 7 and 10 and 15 |

Additional records identified through other sources = 3

138 Records excluded by title / abstract:

(1) were not relevant to NCD or healthcare;

(2) looked at a different population;

(3) focused on health topic unrelated to NCD care.

24 Full-text articles excluded:

(1) measured prevalence or treatment of NCDs only;

(2) looked at barriers to healthcare outside Syria only;

(3) were opinion pieces / commentaries.

Refugee population studies were excluded if they looked at prevalence or treatment only, or were based on non-Syrian populations. They were included in the review if they looked at care within Syria itself.

Records screened

42 Full-text articles assessed for eligibility

21 studies included

Records identified through database searching = 236

Records after duplicates removed = 180

*Figure 3:* *Diagram showing the articles identified for the literature review, and the number of articles excluded at each stage, adapted from PRISMA (44)*

*Table 2: Table showing the main findings on factors impacting NCD healthcare implementation in Syria*

|  | Pre-conflict | Conflict factors |
| --- | --- | --- |
| Understanding the population | Lack of health information systems and quality data. (34) | Conflict makes assessment difficult. (41) |
| Prioritisation and resource allocation | Decline of investment in social and healthcare. (45)  Lack of interest or national strategy in tackling DM and CVD. (34)  Lack of focus on prevention and challenges from the population to implementing a smoking ban. (34, 35) | Prioritisation of trauma over NCDs (29)  Low prioritisation of palliative care (46) |
| Models of Healthcare | Lack of coordination between primary and secondary care (35) | Health services fragmented and no standardised onward referral pathways to specialist care. (11)  Limited continuity of care due to lack of cancer registry. (46) |
| Service delivery | Concerns around care quality and standardisation. (47)  Lack of standardised protocols (34, 35)  Difficulties in employing trained and skilled staff (34, 35)  Concerns around medicine quality (34) | Facilities were too busy or destroyed (48) and a lack of security of healthcare structures (29, 41, 49)  Lack of trained HCW including doctors. (29, 41, 47-49)  Barriers to care reported as out-of-stock medications (48, 50). Lack of medications and resources in part due to decline in in-country production (29, 49) and due to direct sanctions. (9)  Difficulties of a mobile rather than camp-based population (49) |
| Collaboration and governance |  | Challenges in cooperation and coordination (47)  Governance structures “decentralised and fragmented”, and although local governance invaluable in coordinating health services, variable quality. (29)  Politicisation of health, where combatants were prioritised over civilians; doctors were targeted; provision of healthcare was political; and inequity of care. (41)  One paper concluded: “change in this system happened mostly because of political decision, that rarely reflected performance metrics, and health indicators were unreliable and part of the big political propaganda game”. (34) |
