# Supplementary material for: What influenced provision of non-communicable disease healthcare in the Syrian conflict, from policy to implementation? A qualitative study
Source: Confl Health. 2018 Nov 12;12:45. doi: 10.1186/s13031-018-0178-5 (PMC6233508; doi:10.1186/s13031-018-0178-5)
Supplement: Supplementary file 2 — Table S3. Overview of participants interviewed. (DOCX 16 kb) [file 13031_2018_178_MOESM2_ESM.docx]

*Table S3: Overview of participants*

| **Type** | **Current / previous position** | **HCW** | **Worked within Syria pre-conflict** |
| --- | --- | --- | --- |
| **Local†: Syria-related experience gained mainly through work with local organisations** | Currently employed by local organisation | No | Yes |
|  | Currently employed by local organisation | Yes | Yes |
|  | Currently employed by local organisation | Yes | Yes |
|  | Currently employed by iNGO, previously employed by local organisation | Yes | Yes |
|  | Previously employed by local organisation and iNGO | Yes | Yes |
| **International: principal experience at iNGOs** | Currently employed by iNGO, worked remotely | Yes | No |
|  | Currently employed by iNGO, worked remotely | Yes | No |
|  | Currently employed by iNGO, worked remotely | Yes | No |
|  | Currently employed by iNGO, worked within Syria | Yes | No |
|  | Currently employed by iNGO, worked remotely | Yes | No |
|  | Currently employed by iNGO | Yes | Yes |
| **WHO: principal experience at WHO** | WHO | Yes | No |
|  | WHO | Yes | No |
|  | WHO | Yes | No |

†Local or international does not reflect citizenship, but rather experience.
